# Relative telomere lengths in tumor and normal mucosa are related to disease progression and chromosome instability profiles in colorectal cancer

**DOI:** 10.18632/oncotarget.9015

**Published:** 2016-04-26

**Authors:** Nirosha Suraweera, Dmitri Mouradov, Shan Li, Robert N. Jorissen, Debbie Hampson, Anil Ghosh, Neel Sengupta, Mohamed Thaha, Shafi Ahmed, Michael Kirwan, Floor Aleva, David Propper, Roger M. Feakins, Tom Vulliamy, Ngaire J. Elwood, Pei Tian, Robyn L. Ward, Nicholas J. Hawkins, Zheng-Zhou Xu, Peter L. Molloy, Ian T. Jones, Matthew Croxford, Peter Gibbs, Andrew Silver, Oliver M. Sieber

**Affiliations:** ^1^ Centre for Digestive Diseases, Blizard Institute, Barts and The London School of Medicine and Dentistry, Whitechapel, London, UK; ^2^ Systems Biology and Personalised Medicine Division, The Walter and Eliza Hall Institute of Medial Research, Parkville, Victoria, Australia; ^3^ Department of Medical Biology, The University of Melbourne, Parkville, Victoria, Australia; ^4^ Academic Surgical Unit, The Royal London Hospital, Whitechapel, London, UK; ^5^ Centre for Paediatrics, Blizard Institute, Barts and The London School of Medicine and Dentistry, Whitechapel, London, UK; ^6^ Department of Medical Oncology, St Bartholomew's Hospital, Little Britain, London, UK; ^7^ Department of Pathology, The Royal London Hospital, Whitechapel, London, UK; ^8^ Centre for Genomics and Child Health, Blizard Institute, Barts and The London School of Medicine and Dentistry, Whitechapel, London, UK; ^9^ Cord Blood Research, Murdoch Children's Research Institute, Melbourne, Australia; ^10^ Department of Paediatrics, University of Melbourne, Melbourne, Australia; ^11^ The University of Queensland, Brisbane, Queensland, Australia; ^12^ School of Medicine, The University of Queensland, Brisbane, Queensland, Australia; ^13^ CSIRO Preventative Health Flagship, North Ryde, NSW, Australia; ^14^ Department of Colorectal Surgery, Royal Melbourne Hospital, Parkville, Victoria, Australia; ^15^ Department of Surgery, The University of Melbourne, Parkville, Victoria, Australia; ^16^ Department of Colorectal Surgery, Western Hospital, Footscray, Victoria, Australia; ^17^ Department of Medical Oncology, Royal Melbourne Hospital, Parkville, Victoria, Australia; ^18^ School of Biomedical Sciences, Monash University, Victoria, Australia

**Keywords:** telomere length, colorectal cancer, chromosome instability, prognosis

## Abstract

Telomeric dysfunction is linked to colorectal cancer (CRC) initiation. However, the relationship of normal tissue and tumor telomere lengths with CRC progression, molecular features and prognosis is unclear. Here, we measured relative telomere length (RTL) by real-time quantitative PCR in 90 adenomas (aRTL), 419 stage I-IV CRCs (cRTL) and adjacent normal mucosa (nRTL). Age-adjusted RTL was analyzed against germline variants in telomere biology genes, chromosome instability (CIN), microsatellite instability (MSI), CpG island methylator phenotype (CIMP), *TP53*, *KRAS*, *BRAF* mutations and clinical outcomes. In 509 adenoma or CRC patients, nRTL decreased with advancing age. Female gender, proximal location and the *TERT* rs2736100 G allele were independently associated with longer age-adjusted nRTL. Adenomas and carcinomas exhibited telomere shortening in 79% and 67% and lengthening in 7% and 15% of cases. Age-adjusted nRTL and cRTL were independently associated with tumor stage, decreasing from adenoma to stage III and leveling out or increasing from stage III to IV, respectively. Cancer MSI, CIMP, *TP53*, *KRAS* and *BRAF* status were not related to nRTL or cRTL. Near-tetraploid CRCs exhibited significantly longer cRTLs than CIN- and aneuploidy CRCs, while cRTL was significantly shorter in CRCs with larger numbers of chromosome breaks. Age-adjusted nRTL, cRTL or cRTL:nRTL ratios were not associated with disease-free or overall survival in stage II/III CRC. Taken together, our data show that both normal mucosa and tumor RTL are independently associated with CRC progression, and highlight divergent associations of CRC telomere length with tumor CIN profiles.

## INTRODUCTION

Telomeres are specialized structures composed of (TTAGGG)n tandem nucleotide repeats and protein that cap at the ends of linear eukaryotic chromosomes. They have an essential role in maintaining genomic integrity by protecting chromosomes from end-to-end fusion and exonucleolytic degradation [1]. In normal human somatic cells, telomeres have an average length of 5 to 15 kilobases and shorten by ~30 to 200 base pairs every cell division due to the incomplete semi-conservative nature of DNA replication, the so-called end-replication problem [2]. In addition to the mitotic replication rate, endogenous and exogenous factors such as gender, oxidative stress, smoking and obesity contribute to the rate of telomere attrition [3]. With continuous shortening, telomeres eventually reach a critical length that triggers the activation of the p53 and/or RB tumor suppressor pathways and provides the signal for replicative senescence and apoptosis [4]. Cells that aberrantly bypass senescence and continue to divide suffer telomere attrition culminating in chromosome breakage-fusion-bridge (BFB) cycles and genome instability [5]. In immortal cells, telomere shortening is ultimately arrested either by the inappropriate activation of telomerase, a ribonucleoprotein complex consisting of telomerase reverse transcriptase (TERT) and an internal RNA component (TERC), or less commonly by the recombination-mediated alternative lengthening of telomeres (ALT) pathway [6, 7]. Telomere dysfunction is considered a major contributor to cancer development. Telomeres in human cancers are often shorter or elongated as compared to the surrounding normal tissue, telomere attrition and BFB results in numerical and structural chromosomal changes, and tumors frequently exhibit aberrant telomerase activation or ALT [5–7]. Accordingly, mouse models of excessive telomere shortening display increased tumor risk [8].

In human colorectal cancer (CRC), 39% to 97% (mean = 72%) of carcinomas have been reported to exhibit shorter telomeres as compared to adjacent normal mucosa, with the large range of estimates at least partly reflecting differences in analytical methods and classification cut-offs used [9–15]. Telomere shortening is considered an early event in colorectal tumorigenesis, with decreased telomere lengths reported in up to 64% of adenomas [10, 16, 17].

The role of telomere dysfunction in CRC progression remains controversial. Some studies report that telomeres are shorter in carcinomas as compared to adenomas [9, 10, 18], while others have observed no difference or opposite results [19, 20]. Among carcinomas, several investigators have identified that telomeres are longer in more advanced cases, and have suggested this may be explained by progressive re-activation of telomerase or ALT [9, 11, 12, 20], Others have not confirmed a correlation between cancer telomere length and disease stage [13, 14]. Inconclusive findings exist for associations between cancer telomere length and tumor location [9, 11–14] or differentiation [12, 14].

Limited data are available on the relationship between cancer telomere length and clinical outcomes for early-stage CRC. Three studies have identified longer cancer telomere length or higher cancer to non-cancer tissue telomere length ratios as predictors of poor prognosis [11, 12, 14], although results for disease-free and overall survival were not always consistent. Another has reported opposite results, with shorter telomeres associated with worse survival [9].

Few studies have examined the relationship between telomere length and chromosome instability (CIN) profiles in CRC, or investigated other global molecular phenotypes such as microsatellite instability (MSI+) – where there is a propensity to insertion and deletion mutations at simple tandem repeats due to defective DNA mismatch repair (MMR) – or CpG island methylator phenotype (CIMP+). Consistent with a role of telomere dysfunction in driving CIN+, telomere shortening in colorectal polyps from patients with familial adenomatous polyposis has recently been correlated with large-scale genomic rearrangements [17]. In a study of patients with MSI- rectal cancers shorter tumor telomeres were similarly associated with CIN+ and activation of telomerase [15]. In contrast, MSI-/CIN- rectal cancers were found to have longer telomeres than MSI-/CIN+ rectal cancers and to exhibit ALT rather than telomerase activation [15]. Two studies have reported that MSI+ cancers have shorter telomeres than MSI- cancers [13, 21]. Limited data are available on the association between telomere length and somatic mutation profiles such as changes in *TP53*, *KRAS* and *BRAF* [22, 23].

Twin studies and population-based surveys have established that normal telomere length is largely genetically determined [24]. Accordingly, telomere length has been linked to common variants in multiple genes related to telomere biology such as *TERC*, *TERT*, *MEN1*, *OBFC1 RECQL5* and *RTEL1* [25–27]. Telomere length of peripheral blood leukocytes (PBL) and variants in *TERT* and *TERC* have been associated with CRC susceptibility, with evidence that both shorter and longer telomere lengths may play a role [28–30]. One study has identified PBL telomere length as an independent prognostic marker for CRC [31].

Here, we analyzed 509 patients with colorectal adenoma or carcinoma to define the major clinical and germline modifiers associated with RTL in normal colorectal mucosa, and to clarify the respective contributions of normal and tumor RTL to CRC initiation and progression. We further investigated the relationships of normal and tumor RTL with somatic mutation, CIMP, MSI and CIN profiles, and evaluated their respective prognostic value in stage II/III CRC.

## RESULTS

### RTL of cancer and normal mucosa in patients with CRC

Four hundred and nineteen patients with stage I-IV CRCs were screened for RTL of cancer (cRTL) and histologically normal adjacent mucosa (nRTL), MSI, and mutations in *KRAS*, *BRAF*, *PIK3CA* and *TP53*. CIMP status was analyzed in 382 cancers. SNP microarray data, available on 349 matched tumor and normal samples [30], were analyzed for tumor CIN profiles and germline variants in 15 genes previously associated with telomere length in the population-based studies (*ACYP2*, *BCL2L1*, *CTC1*, *CXCR4*, *MEN1*, *MRE11A*, *NAF1*, *OBFC1*, *RECQL5*, *RTEL1*, *TERC*, *TERT*, *TNKS*, *ZNF208* and *ZNF676*) (Supplementary Table S1).

Consistent with other studies, mutations in *KRAS* were detected in 34.4% (144/419), *BRAF* in 11.2% (47/419), *PIK3CA* in 14.1% (59/419) and *TP53* in 55.0% (229/416) of cases. MSI+ and CIN+ was identified in 17.2% (72/419) and 73.6% (257/349) of cancers, respectively. Among CIN+ cancers, 16.0% (41/257) were near-tetraploid and 84.0% (216/257) aneuploidy, with the number of chromosome breaks ranging from 0 to 110 (mean=18) (Figure 3A). 19.6% (75/382) of cancers were CIMP+. MSI+ and CIN+ showed a strong inverse association (OR=0.05), and *KRAS* and *BRAF* mutations were nearly mutually exclusive (OR=0.03).

RTL measured using the multiplex quantitative polymerase chain reaction (qPCR) method developed by Cawthon [32] has previously been shown to highly correlate with absolute telomere length measurements in tumor and normal samples as determined by Southern blotting [13]. Accordingly, we found that copy number of the human beta globin gene, used as internal assay control, was highly correlated with total chromosome number in our tumors as determined from SNP array data (r=0.86, P<0.001; Supplementary Figure S1). The inter-assay coefficient of variability (CV) for qPCR repeat assays was 7.5% for normal and 8.4% for tumor samples (Supplementary Figure S2). Matched tumor and normal samples were analyzed in the same qPCR plate together with references cell line samples with established short telomere length of 3.76 kb (TF-1: human hematopoietic progenitor cell line [33]) and long telomere lengths of 9.92 kb (TF-1/TI2G: TF-1 cell line with retroviral overexpression of hTERT [33]) which were readily distinguished (TF1: RTL mean=0.73, SD=0.13; TF-1/TI2G: RTL mean=3.06, SD=0.37; Supplementary Figure S3). As anticipated, nRTL decreased significantly with older age (P<0.001), and normal and tumor RTL measurements were age-adjusted accordingly using residuals as described by Robles-Espinoza *et al.* (Supplementary Figure S4) [34].

**Figure 1:**
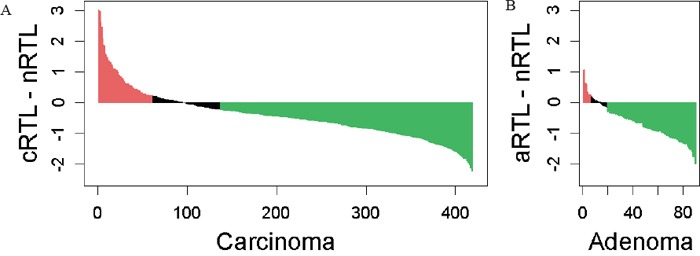
Differences in age-adjusted RTLs between tumor and normal colorectal mucosa for **A.** carcinoma and **B.** adenoma patients Considering 2x the mean standard deviation of nRTL repeat measurements as a cut-off, 67% of carcinomas and 79% of adenomas show telomere shortening (green bars), and 15% of carcinomas and 7% of adenomas show telomere lengthening (red bars).

Age-adjusted telomere length in cancers was significantly shorter as compared to adjacent mucosa, with a cRTL mean of −0.06 (SD=0.51) and an nRTL mean of −0.47 (SD=0.69), respectively (P<0.001). Considering 2x the mean standard deviation of nRTL repeat measurements (2xNSD) as cut-off, 67.3% (282/419) of cancers exhibited telomere shortening and 14.6% (61/419) telomere lengthening, consistent with previous reports evaluating absolute telomere length (Figure 1) [9–15].

### RTL of normal colorectal mucosa is related to patient baseline characteristics and germline variants in telomere biology genes

We tested whether age-adjusted nRTL in histologically normal colorectal mucosa from CRC patients was related to previously implicated baseline clinical and germline modifiers by first considering 28 common variants linked to telomere length in the general population (Supplementary Table S1). In our cohort, the *TERT2* intron 2 variant rs2736100 was significantly associated with nRTL (FDR-adjusted P<0.037), with the G allele linked to longer telomere length consistent with previous reports [30]. Considering patient gender, site of tissue sampling and rs2736100 status in multivariate analysis, female gender, proximal location and rs2736100 G allele were independently associated with longer age-adjusted nRTL (P<0.049 for all comparisons, Table 1A). For the subset of individuals for whom BMI and smoking history details were available (n=229), these variables were not correlated with nRTL (multivariate P>0.417 for both comparisons, Table 1B).

**Table 1:** Characteristics of patients with CRC according to age-adjusted RTL of normal mucosa (nRTL)

A. Clinical Features	All	nRTL	*P*	*P*
		Mean±SD	(Uni.)	(Multi.)
Gender	n=419			
Male	223	−0.13 ± 0.46		
Female	196	0.02 ± 0.54	0.002*	0.002*
Site	n=419			
Right	195	0.00 ± 0.51		
Left	142	−0.11 ± 0.53	0.052	0.049*
Rectum	82	−0.10 ± 0.44	0.129	0.280
rs2736100	n=389			
TT	111	−0.15 ± 0.43		
TG	201	−0.08 ± 0.53		
GG	77	0.13 ± 0.55	<0.001*	<0.001*
**B. Lifestyle factors**					
BMI	n=229			
Mean± SD	27.6 ± 5	-	0.522	0.417
Median	27.7			
Range	15.2 to 43.1			
Smoking	n=324			
No	169	−0.08 ± 0.51		
Yes	155	−0.12 ± 0.53	0.446	0.723

Abbreviations: A. Baseline clinical features and rs2736100 genotype, and B. lifestyle factors.

Uni. = univariate; Multi. = multivariate;

* P<0.05.

### RTL of normal colorectal mucosa is associated with CRC stage at presentation

**Figure 2:**
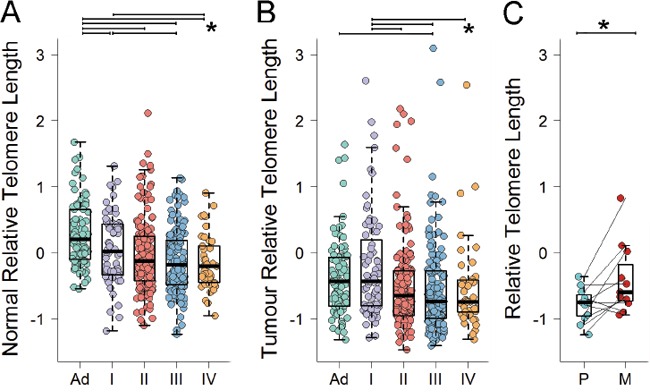
Age-adjusted RTL for **A.** normal mucosa by stage of disease, **B.** tumor by stage of disease, and **C.** matched primary cancer and metastasis pairs Pairwise differences with P<0.05 are indicated.

We next investigated the relationship between age-adjusted nRTL and the pathological and molecular features of the associated cancer (Table 2). In multivariate analysis including gender, location and rs2736100 genotype, nRTL was progressively shorter with increasing tumor stage at presentation for stages I to III, (nRTL mean±SD: stage I 0.07±0.51, stage II −0.02±0.55, stage III −0.13±0.47; P=0.005, Mann-Kendall trend test), but was similar between stages III and IV (nRTL mean±SD: stage III −0.13±0.47, stage IV −0.15±0.42; P=0.387) (Figure 2A). No relationship was detected between nRTL and tumor differentiation (multivariate P=0.760). CRC molecular features including MSI, CIN profile, number of chromosome breaks, CIMP and mutation in *BRAF*, *KRAS* and *TP53* showed no associations with nRTL (multivariate P>0.057 for all comparisons).

**Table 2:** Clinicopathological and molecular characteristics of patients with CRC according to age-adjusted RTL of normal mucosa (nRTL), cancer (cRTL) and cRTL/nRTL ratio

Clinical Features	All	nRTL	*P*	*P*	cRTL	*P*	*P*	cRTL/nRTL	*P*	*P*
		Mean±SD	(Uni.)	(Multi.)	Mean±SD	(Uni.)	(Multi.)	Mean±SD	(Uni.)	(Multi.)
Stage	n=419									
I (Ref.)	71	0.07 ± 0.51			−0.21 ± 0.80			−0.45 ± 0.85		
II	155	−0.02 ± 0.55	0.213	0.090	−0.51 ± 0.66	0.003*	0.250	−0.76 ± 1.04	0.027*	0.355
III	151	−0.13 ± 0.47	0.007*	0.005*	−0.56 ± 0.65	<0.001	0.007*	−0.72 ± 0.96	0.059	0.165
IV	42	−0.15 ± 0.42	0.030*	0.002	−0.54 ± 0.70	0.015*	0.600	−0.66 ± 0.92	0.271	0.596
II (Ref.)	155	−0.02 ± 0.55			−0.51 ± 0.66			−0.76 ± 1.04		
III	151	−0.13 ± 0.47	0.069	0.074	−0.56 ± 0.65	0.486	0.015*	−0.72 ± 0.96	0.687	0.455
IV	42	−0.15 ± 0.42	0.158	0.036*	−0.54 ± 0.70	0.806	0.602	−0.66 ± 0.92	0.548	0.108
III (Ref.)	151	−0.13 ± 0.47			−0.56 ± 0.65			−0.72 ± 0.96		
IV	42	−0.15 ± 0.42	0.827	0.387	−0.54 ± 0.70	0.832	0.027*	−0.66 ± 0.92	0.738	0.033*
Differentiation	n=407									
Well/Moderate	321	−0.05 ± 0.52			−0.48 ± 0.70			−0.69 ± 0.98		
Poor	86	−0.13 ± 0.44	0.182	0.760	−0.48 ± 0.67	0.977	0.225	−0.62 ± 0.94	0.560	0.633
**Molecular Features**										
MSI status	n=419									
Stable	347	−0.06 ± 0.50			−0.49 ± 0.68			−0.69 ± 0.97		
Unstable	72	−0.04 ± 0.54	0.740	0.106	−0.44 ± 0.74	0.557	0.703	−0.64 ± 0.98	0.674	0.141
Ploidy status	n=403									
CIN- (Ref.)	92	−0.08 ± 0.55			−0.62 ± 0.60			−0.84 ± 1.03		
Aneuploid	216	−0.06 ± 0.48	0.726	0.986	−0.56 ± 0.64	0.396	0.207	−0.81 ± 0.98	0.801	0.211
Near-tetraploid	41	0.02 ± 0.49	0.292	0.898	−0.39 ± 0.61	0.042*	0.015*	−0.57 ± 0.82	0.139	0.025*
Aneuploid (Ref.)	216	−0.06 ± 0.48			−0.56 ± 0.64			−0.81 ± 0.98		
Near-tetraploid	41	0.02 ± 0.49	0.366	0.853	−0.39 ± 0.61	0.105	0.046*	−0.57 ± 0.82	0.148	0.079
Chr. breaks	n=349									
Mean± SD	17.6 ± 19.1	-	0.170	0.191	-	0.037*	0.009*	-	0.128	0.057
Median	10									
Range	0 to 110									
CIMP status	n=382									
CIMP-	307	−0.10 ± 0.48			−0.53 ± 0.63			−0.68 ± 0.93		
CIMP+	75	−0.02 ± 0.56	0.204	0.886	−0.41 ± 0.83	0.169	0.946	−0.66 ± 1.06	0.876	0.835
*TP53*	n=416									
Wild-type	187	−0.05 ± 0.52			−0.52 ± 0.65			−0.74 ± 0.96		
Mutated	229	−0.06 ± 0.50	0.751	0.993	−0.46 ± 0.73	0.375	0.115	−0.64 ± 0.98	0.294	0.226
*BRAF*	n=419									
Wild-type	372	−0.06 ± 0.50			−0.49 ± 0.67			−0.69 ± 0.95		
Mutated	47	−0.08 ± 0.58	0.813	0.861	−0.40 ± 0.87	0.411	0.667	−0.64 ± 1.15	0.753	0.996
*KRAS*	n=419									
Wild-type	275	−0.03 ± 0.54			−0.42 ± 0.75			−0.64 ± 1.00		
Mutated	144	−0.12 ± 0.45	0.090	0.057	−0.59 ± 0.56	0.015*	0.142	−0.75 ± 0.91	0.276	0.941

Abbreviations: P-values are for multivariate analysis additionally adjusted for gender, location and rs2736100 genotype.

Uni. = univariate; Multi. = multivariate;

* P<0.05.

### RTL of CRC varies with disease stage and is related to tumor CIN profile

Clinicopathological and molecular correlates of cancer telomere length were evaluated overall (age-adjusted cRTL) and as a change from normal telomere length (age-adjusted cRTL:nRTL ratio) in multivariate analysis (Table 2). As observed for nRTL, shorter cRTL was related to advanced tumor stage decreasing from stage I to III (cRTL mean±SD: stage I −0.21±0.80, stage II −0.51±0.66, stage III −0.56±0.65; P=0.001, Mann-Kendall trend test). cRTL showed evidence of a modest increase from stage III to IV (cRTL mean±SD: stage III −0.56±0.65, stage IV −0.54±0.70; P=0.027) (Figure 2B). A similar but weaker trend was observed when examining cRTL:nRTL ratios (Table 2). Consistent with the observed increase in cRTL between stage IV and earlier tumor stages, liver metastases available for 11 cases tended to show cRTL lengthening as compared to the matched primary cancers (cRTL mean±SD: primary −0.79±0.26, metastasis −0.41±0.53; P=0.024) (Figure 2C). Notably, nRTL and cRTL were independent predictors of tumor stage (nRTL P=0.013, cRTL P=0.013). No association was detected between cRTL and tumor differentiation (multivariate P=0.225) or location (multivariate P>0.454). BMI and smoking history were not associated with cRTL (multivariate P=0.787 and P=0.521, respectively).

**Figure 3:**
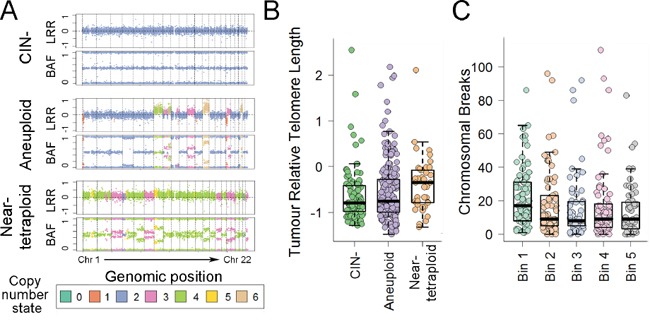
Relationship of age-adjusted RTL with CIN profile in CRC **A.** Representative examples of SNP array data for CIN-, aneuploid and near-tetraploid CRCs. LRR is the log2(observed intensity/reference intensity), while BAF (B Allele Frequency) is the relative contribution of one of the alleles over the total allele signal. **B-C.** Age-adjusted cancer RTL shown by tumor CIN status or the number of chromosome breaks. For chromosomal breaks, the age-adjusted cancer RTL is binned by quintiles.

CRC molecular features including MSI, CIMP and mutations in *BRAF*, *KRAS* and *TP53* were not associated with age-adjusted cRTL or cRTL:nRTL ratios (multivariate P>0.115 for all comparisons) (Table 2). Shorter telomeres in MSI+ cancers have previously been reported in tumors with *TP53* wild-type status [13], but this was not confirmed in a corresponding subset analysis of our cohort (multivariate cRTL P=0.524, cRTL:nRTL ratio P=0.157). However, cRTL showed a divergent association in relation to tumor CIN profiles: cRTL was significantly longer in near-tetraploid tumors as compared to CIN- and aneuploidy tumors (multivariate P<0.046 for both comparisons), whereas cRTL was significantly shorter in tumors with larger numbers of chromosome breaks (multivariate P=0.009) (Table 2, Figure 3). These associations remained significant when restricting the analysis to MSI-tumors (multivariate P≤0.028). A similar but weaker trend was observed when examining cRTL:nRTL ratios (Table 2).

### Adenoma patients tend to have longer normal mucosa RTL and more prevalent tumor telomere shortening than carcinoma patients

To examine the role of normal and tumor telomere length in early carcinogenesis, 90 patients with large (>1cm) tubular or tubulovillous adenomas were screened for RTL of neoplastic (age-adjusted aRTL) and adjacent normal mucosa (age-adjusted nRTL). As anticipated, adenoma telomere length was significantly shorter than matched normal mucosa telomere length, with an aRTL mean of −0.38 (SD=0.57) and nRTL mean of 0.27 (SD=0.48), respectively (P<0.001). Consistent with our observation in CRC patients of shorter normal mucosa telomere length in individuals with more advanced tumors, nRTL in adenoma patients was significantly longer than nRTL in CRC patients adjusting for gender and site (multivariate P<0.001, Figure 2A).

78.8% (71/90) of adenomas exhibited telomere shortening as compared to matched normal mucosa using a 2xNSD cut-off, a higher proportion than among carcinoma cases (67.3% (282/419); P=0.032). However aRTL was overall longer than cRTL (mean±SD: aRTL −0.38±0.57 cRTL −0.47±0.69; P=0.012). In contrast, telomere lengthening appeared less prevalent in adenomas (6.7%, 6/90) than carcinomas (14.6%, 61/419), although this did not reach statistical significance (P=0.057).

### No prognostic value of normal or cancer RTL in stage II/III CRC

We tested whether normal or cancer RTL was associated with prognosis in patients with stage II/III CRC, adjusting for gender, age, tumor site, stage, differentiation and adjuvant treatment. Outcome information was available for 281 patients for OS with a median duration of follow-up of 45.2 months, and for 246 patients for DFS with a median duration of follow-up of 41.6 months.

DFS and OS were similar irrespective of whether age-adjusted nRTL or cRTL were considered (multivariate P≥0.411 for all comparisons, Table 3). Likewise, age-adjusted cRTL:nRTL ratio was not associated with differential outcomes (multivariate P≥0.451). As anticipated, tumor stage and differentiation were independent prognostic factors for DFS, and patient age, gender, tumor stage and differentiation were independent prognostic factors for OS (Table 3). Similar results were obtained when additionally adjusting for tumor MSI and/or CIN status (data not shown).

**Table 3:** Multivariate Cox proportional-hazards analysis of A. disease-free survival and B. overall survival for patients with resected stage II/III CRC according to age-adjusted RTL of normal mucosa (nRTL), cancer (cRTL) and cRTL/nRTL ratio

A. Disease-free survival		nRTL		cRTL		cRTL/nRTL	
	n	HR (95% CI)	*P*	HR (95% CI)	*P*	HR (95% CI)	*P*
Relative telomere length							
T/S ratio	246	0.77 (0.42 - 1.42)	0.411	0.82 (0.47 - 1.45)	0.502	0.89 (0.66 - 1.20)	0.451
Age							
Years	246	0.99 (0.96 - 1.02)	0.374	0.99 (0.96 - 1.02)	0.483	0.99 (0.96 - 1.02)	0.469
Gender							
Male	124	1 (Reference)		1 (Reference)		1 (Reference)	
Female	122	0.61 (0.35 - 1.07)	0.087	0.62 (0.35 - 1.10)	0.102	0.61 (0.35 - 1.08)	0.089
Tumor site							
Proximal colon	116	1 (Reference)		1 (Reference)		1 (Reference)	
Distal colon	94	0.80 (0.42 - 1.51)	0.486	0.83 (0.44 - 1.57)	0.564	0.84 (0.44 - 1.58)	0.585
Rectum	36	1.05 (0.49 - 2.25)	0.905	1.05 (0.49 - 2.27)	0.898	1.05 (0.49 - 2.27)	0.900
Tumor stage								
II	130	1 (Reference)		1 (Reference)		1 (Reference)	
III	116	3.06 (1.47 - 6.37)	0.003*	3.11 (1.49 - 6.47)	<0.001*	3.16 (1.52 - 6.59)	0.002*
Differentiation							
Well/Moderate	188	1 (Reference)		1 (Reference)		1 (Reference)	
Poor	58	2.57 (1.48 - 4.46)	0.001*	2.61 (1.51 - 4.54)	<0.001*	2.63 (1.51 - 4.57)	0.001*
Adjuvant chemotherapy							
No	108	1 (Reference)		1 (Reference)		1 (Reference)	
Yes	138	1.51 (0.72 - 3.16)	0.279	1.48 (0.71 - 3.11)	0.299	1.47 (0.70 - 3.10)	0.307
**B. Overall survival**							
Relative telomere length							
T/S ratio	281	0.82 (0.45 - 1.48)	0.506	1.15 (0.77 - 1.74)	0.493	0.99 (0.75 - 1.32)	0.944
Age							
Years	281	1.03 (1.00 - 1.06)	0.026*	1.03 (1.00 - 1.06)	0.035*	1.03 (1.00 - 1.06)	0.028*
Gender							
Male	146	1 (Reference)		1 (Reference)		1 (Reference)	
Female	135	0.57 (0.33 - 0.98)	0.044*	0.56 (0.32 - 0.96)	0.035*	0.57 (0.33 - 0.98)	0.041*
Tumor site							
Proximal colon	132	1 (Reference)		1 (Reference)		1 (Reference)	
Distal colon	101	0.54 (0.29 - 1.02)	0.056	0.56 (0.30 - 1.04)	0.066	0.56 (0.30 - 1.04)	0.067
Rectum	48	0.86 (0.43 - 1.70)	0.660	0.88 (0.44 - 1.76)	0.724	0.87 (0.44 - 1.72)	0.682
Tumor stage							
II	143	1 (Reference)		1 (Reference)		1 (Reference)	
III	138	2.97 (1.60 - 5.52)	0.001*	3.01 (1.62 - 5.58)	<0.001*	3.00 (1.61 - 5.58)	0.001*
Differentiation							
Well/Moderate	215	1 (Reference)		1 (Reference)		1 (Reference)	
Poor	66	2.01 (1.18 - 3.41)	0.010*	2.05 (1.21 - 3.48)	0.007*	2.06 (1.21 - 3.51)	0.007*
Adjuvant chemotherapy							
No	121	1 (Reference)		1 (Reference)		1 (Reference)	
Yes	160	1.10 (0.59 - 2.05)	0.767	1.11 (0.59 - 2.07)	0.750	1.10 (0.59 - 2.06)	0.761

* P<0.05

## DISCUSSION

This study presents the most comprehensive survey to date of age-adjusted normal and tumor RTL in patients with colorectal adenoma or carcinoma and its relationship to clinicopathological characteristics, germline variants in telomere biology genes, tumor molecular features and outcome.

Telomere length is a heritable trait [24], and population-based studies have implicated germline variants in several genes related to telomere biology [25–27]. Considering 28 germline variants reported to date, we validated the role of rs2736100 – localized in *TERT* intron 2 – with the G allele associated with longer RTL in normal mucosa. Notably, the rs2736100 G allele has been linked to a lower risk of developing CRC [30].

Besides genetic factors, gender and other endogenous and exogenous variables may influence age-adjusted normal tissue telomere length including smoking and obesity [3]. In our cohort, females tended to have longer nRTLs than males consistent with population-based studies examining PBLs [3]. Accordingly, estrogen receptor-alpha signaling has been shown to positively regulate *TERT* transcription and telomerase activity [35]. Proximal location was associated with longer nRTL in our cohort, consistent with the higher baseline telomerase reported for this site [36]. We did not find evidence for a contribution of smoking and BMI, although these details were only available for a subset of individuals.

Age-adjusted nRTL was associated with tumor stage at presentation with progressively shorter nRTLs from adenoma to stage III/IV carcinoma patients. Interestingly, telomere length in PBLs has further been reported to be shorter in patients with adenomas than in individuals with normal colonoscopies [37]. While a relationship between normal tissue telomere length and risk of CRC is well documented [28–30], our data suggest that shorter normal telomere length may additionally provide a background for more rapid cancer progression. Telomere shortening in normal epithelium has been implicated as a direct precursor to the structural and numerical chromosome changes characteristic of CRC [22]. In addition, there is evidence that as normal cells with shortened telomeres enter senescence they exhibit a secretory phenotype that can trigger neighboring cells to by-pass the senescence signal, setting the stage for CIN and tumor development [38, 39].

Telomere shortening is thought to be an early event in colorectal tumorigenesis, although frequency estimates for adenomas vary widely [10, 16, 17]. Our observations indicate telomere shortening in ~79% of adenomatous polyps >1cm in size. Some investigators have described telomere lengthening in 14-30% of adenomas [17, 21], but we found telomere lengthening in only 7% of cases. Some of the discrepancies in published results may be due to differences in adenoma sizes and/or histologic subtypes analyzed, sample preparation or methods and cut-offs to classify telomere behavior.

While there is general agreement that telomere dysfunction occurs early in colorectal tumor development, the relationship between telomere length and tumor progression remains controversial. Some studies report that telomeres are shorter in carcinomas as compared to adenomas [9, 10, 18], while others do not [19, 20]. We found a lower prevalence of telomere shortening in CRCs as compared to adenomas, but the extent of telomere shortening was greater in CRC, in particular at later stages. Telomere lengthening appeared more common in carcinomas as compared to adenomas, although this did not reach statistical significance. Some investigators have reported that telomeres are longer in late-stage than early-stage carcinomas [11, 12], while others have found the opposite or no association [9, 13, 14]. We observed that cRTL significantly decreased from stage I to stage III carcinoma, and showed a modest increase to stage IV carcinoma. This was independent of nRTL, and accordingly cRTL:nRTL ratios showed a similar trend. The apparent increase telomere shortening with advanced cancer stage likely reflects the activation of telomerase and/or ALT with tumor progression [9, 13]. Our data did not validate previous suggestions that cancer telomere length is associated with tumor differentiation [12] and tumor location [12, 13].

Telomere dysfunction leads to CIN, but limited data exist on the correlation of telomere length with CIN status in CRC. Telomere shortening and telomerase activation has been linked to CIN+ in MSI- rectal cancers, while MSI- CIN- rectal cancers had longer telomeres and appeared to exhibit ALT [15]. We identified a divergent association of cancer telomere length depending on the tumor CIN profile, with near-tetraploid tumors tending to have longer telomeres, while short cancer telomeres were associated with higher numbers of chromosome breaks. These data are consistent with an increasing body of data suggesting the potential development of two distinct CIN profiles initiated by endoreduplication caused by telomere dysfunction: maintenance of near-tetraploidy related to early telomere stabilization or elongation, potentially mediated by ALT [40, 41], and development of aneuploidy through subsequent BFB cycles eventually rescued by re-activation of telomerase [5, 42].

Previous data have suggested that MSI+ cancers have shorter telomeres than MSI- cancers, perhaps related to DNA-mismatch repair deficiency associated hypermutation at telomeric repeats [13, 21]. This phenotype may be particularly evident for *TP53* wild-type tumors [13]. Our data did not validate this contention, with MSI+ and MSI- cancers showing similar telomere length even when taking *TP53* mutation status into account. Similar results were observed for CIMP-H status, a marker closely related to the presence of MSI.

Loss of p53 pathway function in primary human cells allows cells to bypass the senescence checkpoint associated with telomere attrition [13, 22]. We found no association between *TP53* mutation status and telomere length. p53 is a known negative regulator of hTERT promoter, and mutated p53 may result in hTERT activation counteracting telomere attrition [43]. Some data suggest a role of EGFR signaling in regulating the telomerase complex, perhaps related to response to anti-EGFR antibody therapy [44, 45]. We found no evidence for an association of *KRAS* or *BRAF* mutation with telomere length.

While multiple studies indicate that telomerase activity is associated with prognosis in stage II/III CRC [46], data on the relationship between telomere length measurements and outcome are limited. Longer cancer telomere length or higher cancer to non-cancer tissue telomere length ratios have been associated with poorer prognosis in three reports [11, 12, 14], but another study has found opposite results [9]. Variation in normal tissue telomere length (for PBLs) has further been suggested as a marker of CRC prognosis [31]. In our cohort of patients with stage II/III CRC we found no evidence of normal or cancer RTL associated with disease-free survival or overall survival. Our observation is consistent with emerging data that TERT levels and/or telomerase activity may be better prognostic markers than telomere length [46].

Strengths of our study include the large number of cases, long-term follow-up and the ability to adjust for major confounders. Our survey also has inherent limitations that relate to the applied methods. We used qPCR to measure RTL, rather than absolute telomere length *per se*, because qPCR is the only practical high-throughput method for large-scale epidemiologic studies. However, the RTL qPCR method developed by Cawthon [32] has previously been shown to highly correlate with absolute telomere length measurements in tumor and normal samples by Southern blotting [13], and we confirmed a high correlation of human beta globin gene and total chromosome number in our tumors. RTL values represent an average telomere length measurement across all chromosomes, but it is clearly established that that it is the shortest telomere in a given cell that is responsible for initiating CIN [47]. However, the well-documented association between average telomere length and cancer risk in human and animal models indicates a tractable direct correlation between average and shortest telomere length [28–30]. Only single normal mucosa samples were available per patient in our study, thus whether nRTL measurements in tumor-adjacent mucosa were representative of the entire colorectum or potentially pertained to a field effect – as has been suggested by some investigators [48] - could not be examined. However our normal biopsies showed no evidence of mutations, CIMP or DNA copy number changes.

The development of telomerase inhibitors as experimental cancer treatment highlights the importance of understanding tumor telomere length variability and its relationship to patient characteristics, molecular features and outcome [49]. Our findings identify patient baseline clinical and genetic modifiers of normal mucosa telomere length, and reveal independent contributions of normal and tumor telomere length to disease progression. We further demonstrate divergent associations of cancer telomere length with distinct CIN profiles. Our results do not support a prognostic value of RTL in resected stage II/III CRC.

## MATERIALS AND METHODS

### Patients

A total of 419 patients with CRC and 90 patients with colorectal adenomas were retrieved from biobanks of two centers, The Royal London Hospital in the United Kingdom and The Royal Melbourne Hospital in Australia. Patients with involved resection margins, hereditary CRC syndromes, ulcerative colitis or Crohn's disease-associated CRC were excluded, and tumours were required to have a greater than 70% neoplastic cell content. Among patients with CRC, 71 had stage I, 155 stage II, 151 stage III, and 42 stage IV disease. 195 were proximal colon (cecum to transverse colon), 142 were distal colon (splenic flexure to rectosigmoid) and 82 were rectal cancers. Patient characteristics are summarized in Supplementary Table S2. Paired liver metastases were available for 11 primary cancers. Body mass index, smoking history, clinical, treatment and follow-up data were prospectively recorded. This study was human research ethics committee-approved, and all patients gave informed consent.

### DNA extraction

Fresh-frozen tumor and normal tissues were collected at surgery and macrodissected following histologic review of an H&E-stained section, with tumor areas comprising greater than 70% neoplastic cells (median=80%). Genomic DNA was extracted using the DNAeasy Blood & Tissue DNA Isolation Kit (QIAGEN).

### Microsatellite instability analysis

The Bethesda consensus panel of microsatellite markers (BAT25, BAT26, D2S123, D5S346, and D17S250) was used to determine tumor MSI status. DNA was PCR-amplified for matched tumor and normal samples using fluorescently labeled primers and reaction products were analyzed on a 3130xl Genetic Analyzer (Applied Biosystems). MSI+ was diagnosed if instability was detected at 2 or more markers.

### Mutation detection

DNA sequencing was performed for *KRAS* (codons 12, 13 and 61), *BRAF* (codon 600), *PIK3CA* (exons 9 and 20) and *TP53* (exons 4-9) on a 3730xl DNA Analyzer (Applied Biosystems) using the BigDye Terminator v3.1 Ready Reaction Mix (Applied Biosystems; details available from authors). Detected mutations were confirmed by resequencing of tumor and matched normal DNA from new PCR product.

### CIMP marker analysis

Tumor CIMP status was determined using MethyLight real-time PCR for the 5 marker panel developed by Weisenberger *et al.* (*IGF2*, *SOCS1*, *RUNX3*, *CACNA1G*, and *NGN1*) in the Australian samples [50], and for the 6 marker panel developed by Toyota *et al.* (MINT1, MINT2, MINT31, *p16^INK4a^, p14^ARF^, MLH1*) in the United Kingdom samples as described [51]. Tumors with methylation at 3 or more CIMP markers were classified as CIMP+.

### Genotyping

Genotypes for germline variants associated with telomere length (Supplementary Table S1) in the population were retrieved from existing Illumina Human610-Quad BeadChip data for our carcinoma patients [30]: *ACYP2*, rs11125529 (proxy rs10165485); *BCL2L1*, rs6060627; *CTC1*, rs3027234 (proxy rs11651199); *CXCR4*, rs4452212 (proxy rs10221893); *MEN1*, rs669976, rs509386, rs2957154, rs670358; *MRE11A*, rs12270338, rs13447720; *NAF1*, rs7675998 (proxy rs11100479); *OBCF1*, rs2487999, rs9419958, rs9420907; *RECQL5*, rs820152; *RTEL1*, rs755017 (proxy rs2281929); *TERC*, rs10936599, rs12696304 (proxy rs1997392), rs6793295; *TERT*, rs2736100; *TNKS*, rs11991621, rs12549064, rs10903314, rs6990300, rs11249943, rs17150478; *ZNF208*, rs8105767 (proxy rs7257051); *ZNF676*, rs412658 (proxy rs10419926). SNP array-based genotyping results for rs2736100 have previously been validated using KASPar competitive allele-specific PCR chemistry (KBiosciences Ltd, Hoddesdon UK [30]).

### CIN assessment using SNP microarrays

Tumor CIN status was determined from existing Illumina Human610-Quad BeadChip data on matched tumor and normal DNA using OncoSNP as described previously [52]. Briefly, copy-number for individual autosomes was estimated by calculating the mode of absolute DNA copy-numbers across SNPs, and the total autosome number was determined for each cancer. The number of chromosome breaks was recorded for each tumor. Accuracy of total autosome copy-number estimates has previously been confirmed using 32 CRC cell lines with standard karyotype data [52]. Tumors with ≤3 whole-autosome changes were classified as CIN-, tumors with a modal autosome copy-number of 4 were classified as near-tetraploid, and other tumors with >3 autosome copy-number changes were classified as aneuploid.

### Telomere length analysis

RTL was measured using the monochrome multiplex quantitative polymerase chain reaction (qPCR) method developed by Cawthon [32]. Briefly, telomeric DNA (T) and a single-copy internal control (S) gene (the human beta globin gene) were amplified for each sample on a 7500 Fast Real-time PCR System (Applied Biosystems) on two occasions, RTL was calculated as T/S ratio against a standard curve. Matched tumor and normal samples were analyzed in the same qPCR plate together with references cell line samples with established short telomere length (TF-1: human hematopoietic progenitor cell line, 3.76 Kb) and long telomere lengths(TF-1/TI2G: TF-1 cell line with retroviral overexpression of hTERT, 9.92 kb) (Supplementary Figure S3) [33]. Reference samples were readily distinguishable with a mean RTL of 0.73 (SD=0.13) for TF-1 and 3.06 (SD=0.37) for TF-1/TI2G. RTL measurements across repeat assays were highly consistent (normal, r=0.91; tumor, r=0.96), with an inter-assay coefficient of variability of 7.5% and 8.4% for normal and tumor samples, respectively (Supplementary Figure S2). RTL measurements for repeat assays were averaged. Validity of using the human beta globin gene as internal control (S) gene in aneuploidy tumor samples was confirmed in our SNP array data, exhibiting a high correlation with total autosome number (r=0.86, P<0.001; Supplementary Figure S1).

### Statistical analysis

Statistical analyses were conducted using the statistical computing software R (R Development Core Team, 2011). Univariate analyses for differences between groups used Fisher exact test for categorical variables and Student's t test for continuous variables. For the analyses for germline variants, an additive genotype model was used. Multivariate analyses for association between RTL and clinicopathological and molecular features were conducted using logistic regression. Outcome analyses for patients with resected stage II/III CRC were performed for 5-year disease-free survival (DFS), and overall survival (OS). DFS was defined as time from surgery to the first confirmed relapse, with censoring done when a patient died or was alive without recurrence at last contact. OS was defined as time from surgery to death, with censoring done when a patient was alive at last contact. Survival curves were generated according to the method of Kaplan and Meier. Univariate survival distributions were compared using the log-rank test, and multivariate analyses used Cox proportional hazards models. A two-sided P-value of <0.05 was considered significant.

## SUPPLEMENTARY DATA





## Abbreviations

ACYP2: acylphosphatase 2 muscle type
ALT: alternative lengthening of telomeres
APC: adenomatous polyposis coli
aRTL: adenoma relative telomere length
BCL2L1: BCL2-like 1
BFB: breakage-fusion-bridge
BICD1: bicaudal D homolog 1
BMI: body mass index
BRAF: v-raf murine sarcoma viral oncogene homolog B1
CACNA1G: calcium channel, voltage-dependent, T type, alpha 1G subunit
CDKN2A (p16^INK4a^/p14^ARF^): cyclin-dependent kinase inhibitor 2A
CI: confidence interval
CIMP: CpG island methylator phenotype
CIN: chromosome instability
CRC: colorectal cancer
cRTL: carcinoma relative telomere length
CTC1: CTS telomere maintenance complex component 1
CXCR4: chemokine (C-X-C motif) receptor 4
DFS: disease-free survival
DNA: deoxyribonucleic acid
EGFR: epidermal growth factor receptor
FAP: familial adenomatous polyposis
HR: hazards ratio
IGF2: insulin-like growth factor 2
KRAS: v-Ki-ras2 Kirsten rat sarcoma viral oncogene homolog
MEN1: multiple endocrine neoplasia I
MINT: methylated-in-tumor
MLH1: mutL homolog 1, colon cancer, nonpolyposis type 2 (E. coli)
MMR: DNA mismatch repair
MRE11A: MRE11 homolog A, double strand break repair nuclease
MSH2: mutS homolog 2
MSI: microsatellite instability
NAF1: nuclear assembly factor 1 ribonucleoprotein
NGN1: neurogenin 1
nRTL: normal mucosa relative telomere length
PBL: peripheral blood lymphocyte
OBFC1: oligonucleotide/oligosaccharide-binding fold containing 1
OS: overall survival
PBL: peripheral blood leukocytes
PIK3C3: phosphatidylinositol 3-kinase, catalytic subunit type 3
PIK3CA: phosphatidylinositol-4,5-bisphosphate 3-kinase, catalytic subunit alpha
qPCR: quantitative PCR
RB: retinoblastoma, RECQL5, RecQ helicase-like 5
RTEL1: regulator of telomere elongation helicase 1
RUNX3: runt-related transcription factor 3
S: single-copy internal control gene
SOCS1: suppressor of cytokine signaling 1
T: telomeric DNA
TERC: telomerase RNA component
TERT: telomerase reverse transcriptase
TNKS: tankyrase, TRF1-interacting ankyrin-related ADP-ribose polymerase
TP53: tumor protein 53
ZNF208: zinc finger protein 208
ZNF676: zinc finger protein 676
