# Supplementary material for: Relative telomere lengths in tumor and normal mucosa are related to disease progression and chromosome instability profiles in colorectal cancer
Source: Oncotarget. 2016 Apr 26;7(24):36474–88. doi: 10.18632/oncotarget.9015 (PMC5095014; doi:10.18632/oncotarget.9015)
Supplement: Supplementary file 1 [file oncotarget-07-36474-s001.pdf]

SUPPLEMENTARY DATA

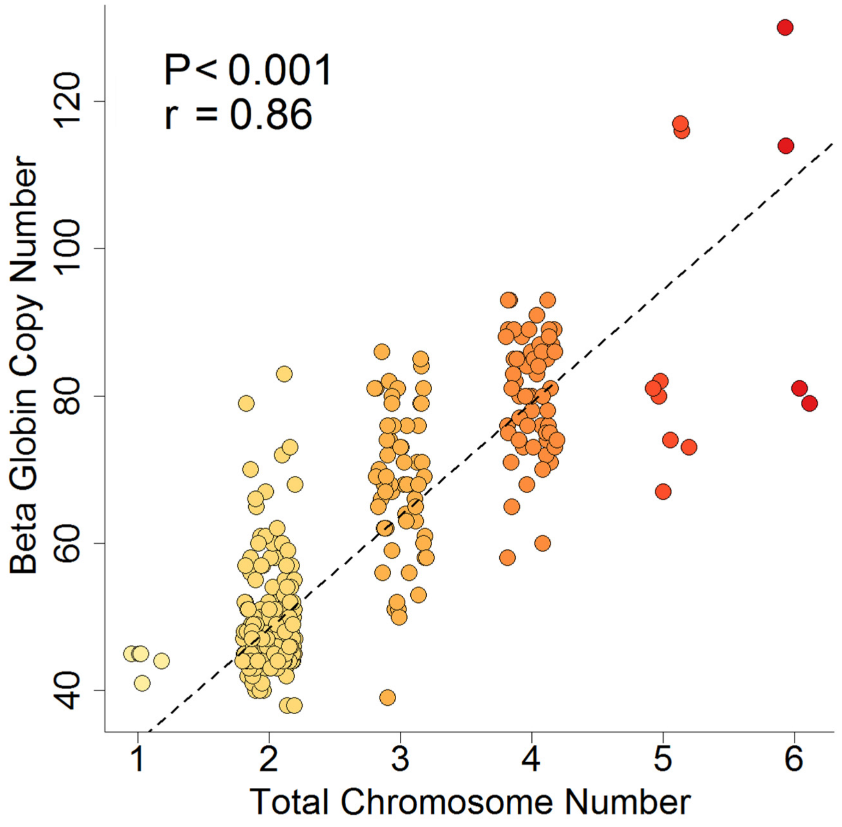

Supplementary Figure S1: Tumor copy number of the human beta globin gene, used as internal control gene in multiplex qPCR assays for relative telomere length, exhibits a high correlation with tumor total chromosome number estimated from SNP array data. Dotted line represents the line of best fit.

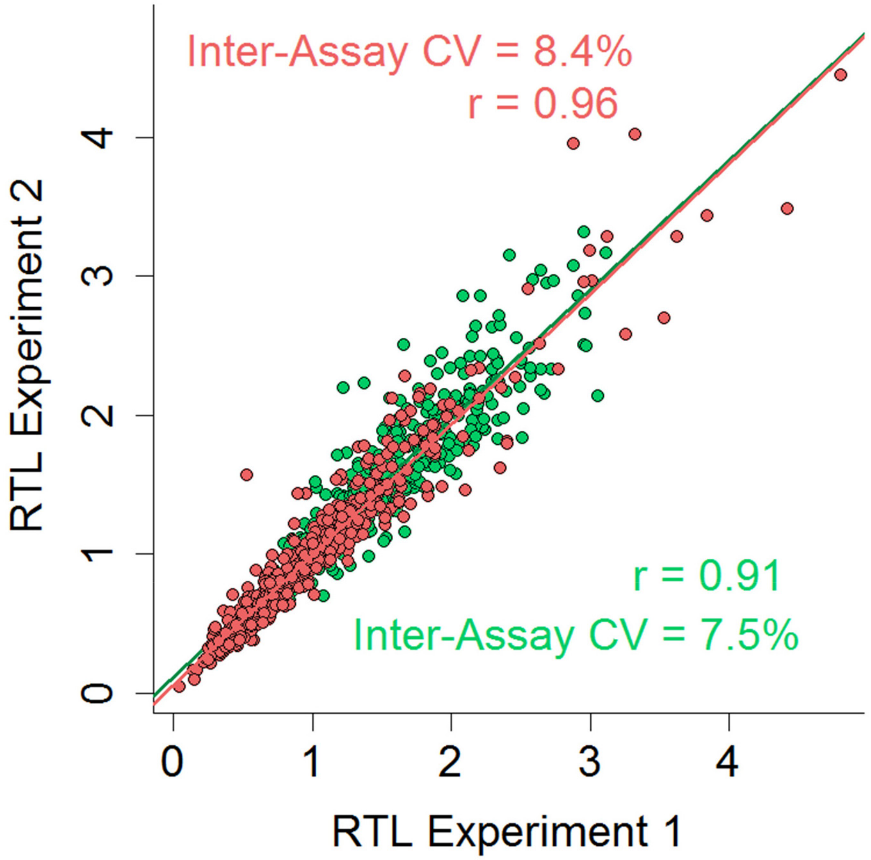

Supplementary Figure S2: Concordance and intra-assay coefficients of variability for replicate RTL measurements (separate plates) for normal mucosa (red) and tumor tissue (green) for patients with colorectal adenoma or carcinoma.

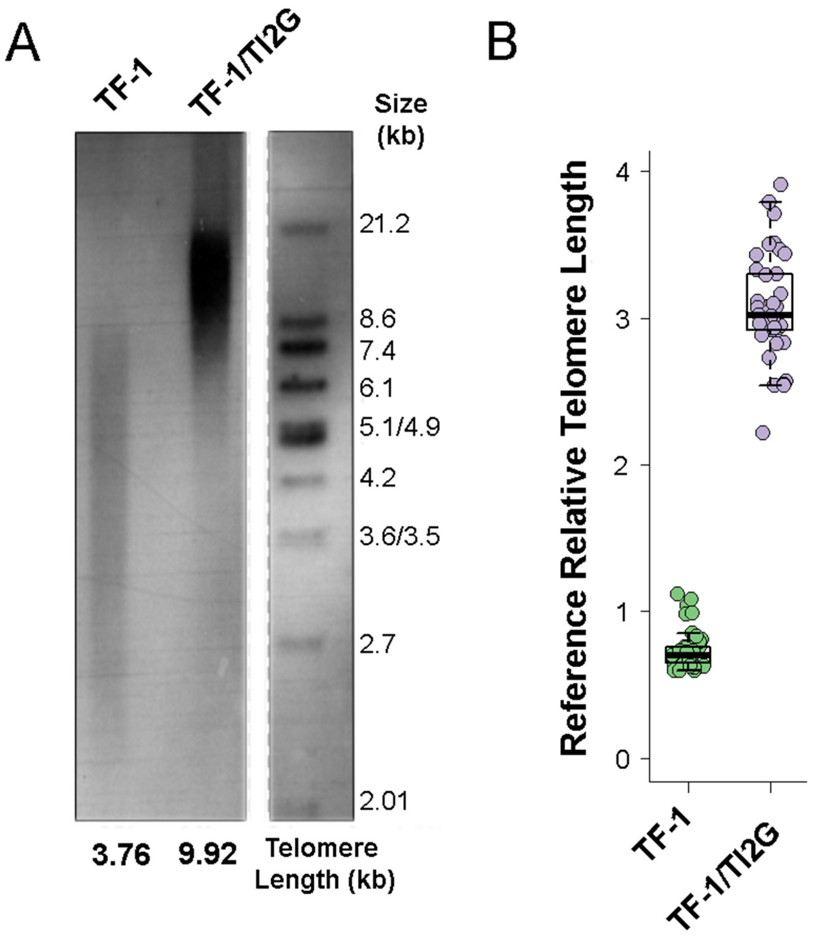

Supplementary Figure S3: Telomere length measurements determined by A. Southern blotting (absolute telomere length) and B. multiplex qPCR (relative telomere length) for parental TF-1 cells and TF1 cells transduced with a retroviral vector overexpressing hTERT (TF1/TI2G).

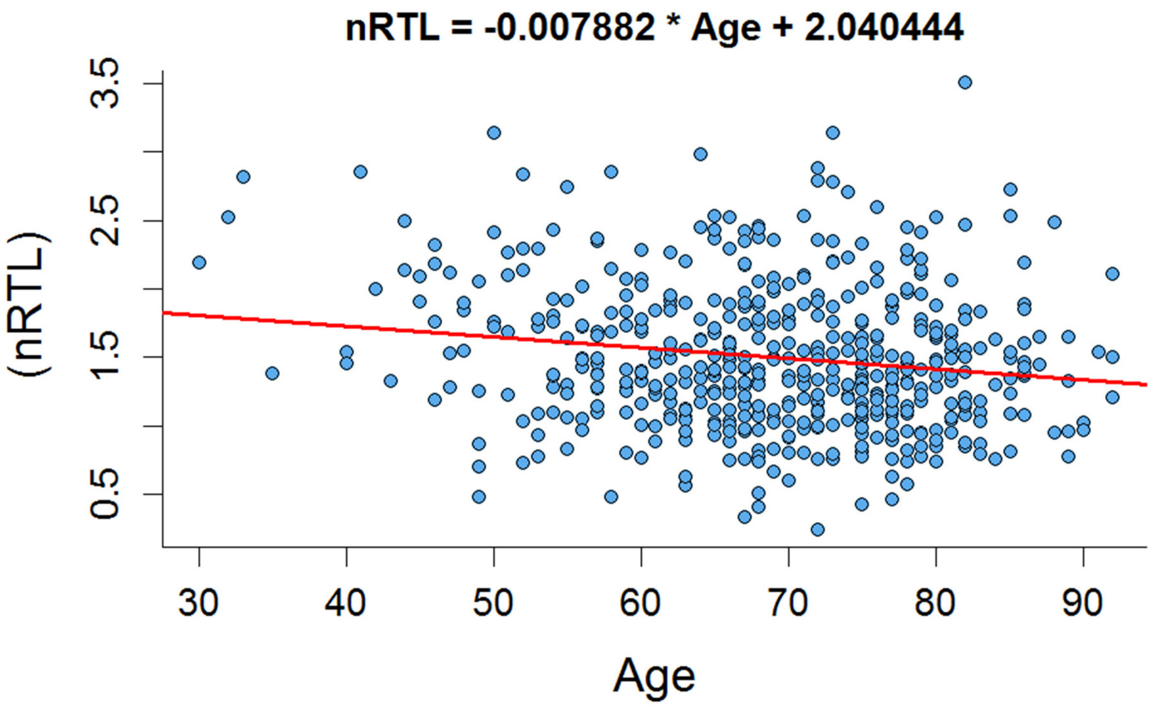

Supplementary Figure S4: Normal mucosa RTL (nRTL) from patients with colorectal adenoma or carcinoma according to age. The linear model used to adjust telomere lengths for age is indicated; residuals were used as the adjusted relative telomere lengths as described in Robles-Espinoza *et al* [1].

Supplementary Table S1: Germline variants in telomere biology related genes according to age-adjusted RTL of normal mucosa (nRTL) in patients with CRC. *P<0.05

See Supplementary File 1

Supplementary Table S2: Clinicopathologic characteristics of A. 419 patients with CRC and B. 90 patients with colorectal adenomas

A. CRC patients	n (%)
**Age**	
Median(Range)	70 (30 to 92)
**Gender**	
Male	223 (53.2)
Female	196 (46.8)
**Site**	
Proximal	195 (46.5)
Distal	142 (33.9)
Rectum	82 (19.6)
**Stage**	
I	71 (16.9)
II	155 (37)
III	151 (36)
IV	42 (10)
**Differentiation**	
Well/Moderate	321 (78.9)
Poor	86 (21.1)
**Adjuvant treatment**	
No	230 (60.1)
Yes	153 (39.9)

B. Adenoma patients	n (%)
**Age**	
Median(Range)	67 (32 to 85)
**Gender**	
Male	46 (51.1)
Female	44 (48.9)
**Site**	
Proximal	52 (58.4)
Distal	17 (19.1)
Rectum	20 (22.5)

REFERENCE

1. Robles-Espinoza CD, Harland M, Ramsay AJ, Aoude LG, Quesada V, Ding Z, Pooley KA, Pritchard AL, Tiffen JC, Petljak M, Palmer JM, Symmons J, Johansson P, Stark MS, Gartside MG, Snowden H, et al. POT1 loss-of-function variants predispose to familial melanoma. Nature genetics. 2014; 46:478-481.
