# Supplementary material for: Relative telomere lengths in tumor and normal mucosa are related to disease progression and chromosome instability profiles in colorectal cancer
Source: Oncotarget. 2016 Apr 26;7(24):36474–88. doi: 10.18632/oncotarget.9015 (PMC5095014; doi:10.18632/oncotarget.9015)
Supplement: Supplementary file 2 [file oncotarget-07-36474-s002.doc]

**SUPPLEMENTARY TABLES AND FIGURES**

**Supplementary Table 1.** Germline variants in telomere biology related genes according to age-adjusted RTL of normal mucosa (nRTL) in patients with CRC. *P<0.05

| **Gene** | **Reference (PMID)** | **SNP** | **Proxy** | **Genotype** | **n** | **nRTL**  **Mean ± SD** | **Unadjusted *P*** | **FDR-adjusted *P*** |
| --- | --- | --- | --- | --- | --- | --- | --- | --- |
| ACYP2 | 23535734 | rs11125529 | rs10165485 | TT | 313 | -0.01 ± 0.49 |  |  |
|  |  |  |  | TC | 100 | -0.06 ± 0.44 |  |  |
|  |  |  |  | CC | 4 | -0.12 ± 0.66 | 0.315 | 0.978 |
| BCL2L1 | 23900074 | rs6060627 | NA | TT | 42 | 0.04 ± 0.51 |  |  |
|  |  |  |  | TC | 176 | -0.06 ± 0.46 |  |  |
|  |  |  |  | CC | 192 | -0.03 ± 0.49 | 0.697 | 0.978 |
| CTC1 | 23001564 | rs3027234 | rs11651199 | AA | 19 | 0.09 ± 0.64 |  |  |
|  |  |  |  | AG | 120 | -0.07 ± 0.43 |  |  |
|  |  |  |  | GG | 271 | -0.03 ± 0.48 | 0.891 | 0.978 |
| CXCR4 | 20421499 | rs4452212 | rs10221893 | TT | 108 | 0.00 ± 0.46 |  |  |
|  |  |  |  | TC | 153 | -0.08 ± 0.47 |  |  |
|  |  |  |  | CC | 149 | -0.01 ± 0.49 | 0.995 | 0.995 |
| MEN1 | 20597107 | rs669976 | NA | TT | 329 | -0.02 ± 0.49 |  |  |
|  |  |  |  | TC | 83 | -0.06 ± 0.46 |  |  |
|  |  |  |  | CC | 5 | 0.23 ± 0.51 | 0.871 | 0.978 |
| MEN1 | 20597107 | rs524386 | NA | TT | 335 | -0.02 ± 0.49 |  |  |
|  |  |  |  | TC | 76 | -0.08 ± 0.46 |  |  |
|  |  |  |  | CC | 6 | 0.26 ± 0.50 | 0.879 | 0.978 |
| MEN1 | 20597107 | rs2957154 | NA | AA | 238 | -0.02 ± 0.49 |  |  |
|  |  |  |  | AG | 156 | -0.04 ± 0.49 |  |  |
|  |  |  |  | GG | 23 | 0.04 ± 0.40 | 0.877 | 0.978 |
| MEN1 | 20597107 | rs670358 | NA | AA | 14 | 0.10 ± 0.57 |  |  |
|  |  |  |  | AG | 81 | -0.08 ± 0.46 |  |  |
|  |  |  |  | GG | 322 | -0.02 ± 0.49 | 0.819 | 0.978 |
| MRE11A | 20597107 | rs12270338 | NA | AA | 16 | 0.20 ± 0.60 |  |  |
|  |  |  |  | AC | 159 | -0.05 ± 0.47 |  |  |
|  |  |  |  | CC | 237 | -0.03 ± 0.48 | 0.485 | 0.978 |
| MRE11A | 20597107 | rs13447720 | NA | AA | 234 | -0.02 ± 0.49 |  |  |
|  |  |  |  | AG | 164 | -0.05 ± 0.47 |  |  |
|  |  |  |  | GG | 19 | 0.14 ± 0.57 | 0.795 | 0.978 |
| NAF1 | 23535734 | rs7675998 | rs11100479 | TT | 251 | -0.05 ± 0.48 |  |  |
|  |  |  |  | TC | 136 | 0.00 ± 0.49 |  |  |
|  |  |  |  | CC | 30 | 0.05 ± 0.43 | 0.158 | 0.918 |
| OBCF1 | 25862531 | rs2487999 | NA | TT | 2 | -0.13 ± 0.02 |  |  |
|  |  |  |  | TC | 78 | 0.06 ± 0.48 |  |  |
|  |  |  |  | CC | 330 | -0.06 ± 0.48 | 0.068 | 0.918 |
| OBFC1 | 20421499 | rs9419958 | NA | TT | 5 | -0.19 ± 0.12 |  |  |
|  |  |  |  | TC | 114 | 0.03 ± 0.48 |  |  |
|  |  |  |  | CC | 297 | -0.04 ± 0.49 | 0.372 | 0.978 |
| OBFC1 | 23535734 | rs9420907 | NA | AA | 298 | -0.04 ± 0.49 |  |  |
|  |  |  |  | AC | 114 | 0.03 ± 0.48 |  |  |
|  |  |  |  | CC | 5 | -0.19 ± 0.12 | 0.360 | 0.978 |
| RECQL5 | 20597107 | rs820152 | NA | TT | 149 | -0.07 ± 0.46 |  |  |
|  |  |  |  | TC | 201 | -0.01 ± 0.49 |  |  |
|  |  |  |  | CC | 67 | 0.04 ± 0.51 | 0.112 | 0.918 |
| RTEL1 | 23535734 | rs755017 | rs2281929 | TT | 310 | -0.04 ± 0.47 |  |  |
|  |  |  |  | TC | 96 | 0.01 ± 0.51 |  |  |
|  |  |  |  | CC | 11 | -0.06 ± 0.59 | 0.542 | 0.978 |
| TERC | 21708826 | rs10936599 | NA | TT | 29 | -0.02 ± 0.57 |  |  |
|  |  |  |  | TC | 161 | -0.08 ± 0.49 |  |  |
|  |  |  |  | CC | 227 | 0.01 ± 0.47 | 0.181 | 0.918 |
| TERC | 20139977 | rs12696304 | rs1997392 | AA | 34 | -0.04 ± 0.56 |  |  |
|  |  |  |  | AG | 177 | -0.06 ± 0.49 |  |  |
|  |  |  |  | GG | 206 | 0.01 ± 0.47 | 0.230 | 0.918 |
| TERC | 24908248 | rs6793295 | NA | TT | 206 | 0.01 ± 0.47 |  |  |
|  |  |  |  | TC | 177 | -0.06 ± 0.49 |  |  |
|  |  |  |  | CC | 34 | -0.04 ± 0.56 | 0.230 | 0.918 |
| TERT | 23535734 | rs2736100 | NA | TT | 106 | -0.10 ± 0.44 |  |  |
|  |  |  |  | TG | 217 | -0.06 ± 0.51 |  |  |
|  |  |  |  | GG | 93 | 0.13 ± 0.45 | 0.001* | 0.037* |
| TNKS | 20597107 | rs11991621 | NA | TT | 17 | -0.15 ± 0.55 |  |  |
|  |  |  |  | TC | 104 | 0.00 ± 0.45 |  |  |
|  |  |  |  | CC | 296 | -0.03 ± 0.49 | 0.685 | 0.978 |
| TNKS | 20597107 | rs12549064 | NA | AA | 287 | -0.03 ± 0.49 |  |  |
|  |  |  |  | AC | 111 | 0.00 ± 0.46 |  |  |
|  |  |  |  | CC | 17 | -0.21 ± 0.51 | 0.514 | 0.978 |
| TNKS | 20597107 | rs10903314 | NA | TT | 27 | -0.10 ± 0.51 |  |  |
|  |  |  |  | TC | 148 | -0.02 ± 0.45 |  |  |
|  |  |  |  | CC | 241 | -0.02 ± 0.50 | 0.582 | 0.978 |
| TNKS | 20597107 | rs6990300 | NA | AA | 187 | -0.03 ± 0.51 |  |  |
|  |  |  |  | AG | 180 | -0.01 ± 0.46 |  |  |
|  |  |  |  | GG | 50 | -0.04 ± 0.47 | 0.884 | 0.978 |
| TNKS | 20597107 | rs11249943 | NA | AA | 271 | -0.03 ± 0.49 |  |  |
|  |  |  |  | AC | 130 | 0.00 ± 0.47 |  |  |
|  |  |  |  | CC | 16 | -0.19 ± 0.53 | 0.66 | 0.978 |
| TNKS | 20597107 | rs17150478 | NA | AA | 289 | -0.04 ± 0.48 |  |  |
|  |  |  |  | AG | 113 | 0.02 ± 0.48 |  |  |
|  |  |  |  | GG | 15 | -0.19 ± 0.48 | 0.926 | 0.978 |
| ZNF208 | 23535734 | rs8105767 | rs7257051 | AA | 180 | -0.02 ± 0.49 |  |  |
|  |  |  |  | AG | 172 | -0.03 ± 0.47 |  |  |
|  |  |  |  | GG | 65 | -0.02 ± 0.49 | 0.943 | 0.978 |
| ZNF676 | 23001564 | rs412658 | rs10419926 | AA | 167 | -0.05 ± 0.48 |  |  |
|  |  |  |  | AG | 168 | -0.02 ± 0.48 |  |  |
|  |  |  |  | GG | 75 | -0.05 ± 0.47 | 0.908 | 0.978 |
